# Dynamical Modeling of the Cell Cycle and Cell Fate Emergence in *Caulobacter crescentus*

**DOI:** 10.1371/journal.pone.0111116

**Published:** 2014-11-04

**Authors:** César Quiñones-Valles, Ismael Sánchez-Osorio, Agustino Martínez-Antonio

**Affiliations:** 1 Engineering and Biomedical Physics Department, Center for Research and Advanced Studies of the National Polytechnic Institute at Monterrey, Apodaca, Nuevo León, México; 2 Genetic Engineering Department, Center for Research and Advanced Studies of the National Polytechnic Institute at Irapuato, Irapuato, Guanajuato, México; University of Erlangen-Nuremberg, Germany

## Abstract

The division of *Caulobacter crescentus*, a model organism for studying cell cycle and differentiation in bacteria, generates two cell types: swarmer and stalked. To complete its cycle, *C. crescentus* must first differentiate from the swarmer to the stalked phenotype. An important regulator involved in this process is CtrA, which operates in a gene regulatory network and coordinates many of the interactions associated to the generation of cellular asymmetry. Gaining insight into how such a differentiation phenomenon arises and how network components interact to bring about cellular behavior and function demands mathematical models and simulations. In this work, we present a dynamical model based on a generalization of the Boolean abstraction of gene expression for a minimal network controlling the cell cycle and asymmetric cell division in *C. crescentus*. This network was constructed from data obtained from an exhaustive search in the literature. The results of the simulations based on our model show a cyclic attractor whose configurations can be made to correspond with the current knowledge of the activity of the regulators participating in the gene network during the cell cycle. Additionally, we found two point attractors that can be interpreted in terms of the network configurations directing the two cell types. The entire network is shown to be operating close to the critical regime, which means that it is robust enough to perturbations on dynamics of the network, but adaptable to environmental changes.

## Introduction

**Figure 1:**
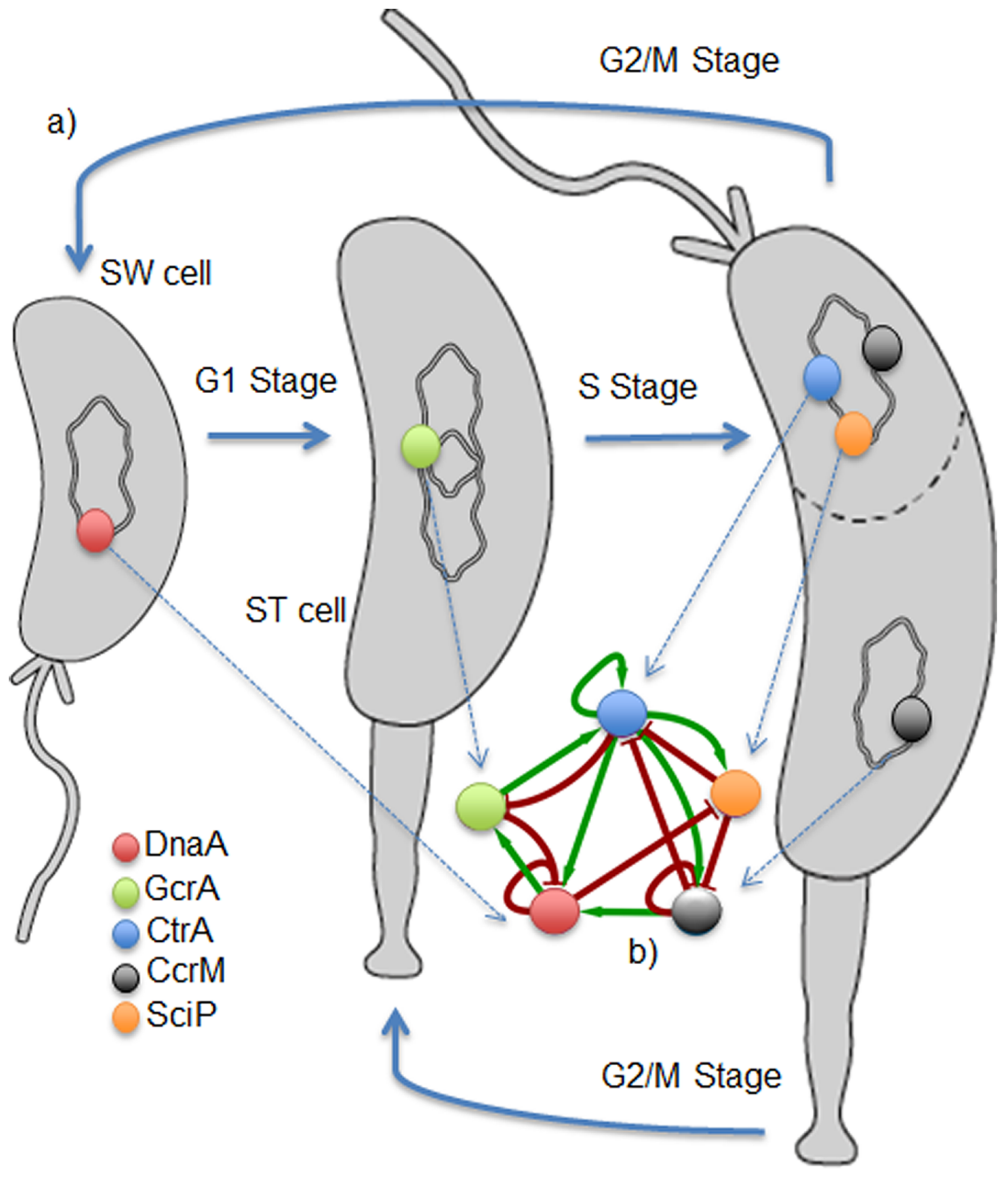
Schematic diagram for the cell cycle in *C. crescentus*. I) Cell cycle stages: The swarmer cell differentiates into the stalked cell in stage G1 (DNA replication initiates mainly due to DnaA) when the stalked cell pre-divides and the chromosomes segregates to each pole (stage S mediated by the action of GcrA). In stage G2/M, CtrA promotes asymmetric cell division and the generation of the two phenotypes (along with SciP, it co-regulates many genes and CcrM full methylates the DNA). II) Regulatory network constituted by the regulators DnaA, GcrA, CtrA, CcrM and Scip, which interact among each other.

*Caulobacter crescentus* is a model organism used to study cell cycle and differentiation in bacteria [1]. It is known that this bacterium differentiates and divides asymmetrically generating two phenotypes: the stalked (ST) and swarmer (SW) cell type [2], [3]. Its cell cycle shares operational similarities with the cell cycle in some eukaryotic organisms [4]. For study purposes, the cycle in this bacterium is divided into three main stages: G1, S and G2/M [5]. G1 is characterized by the differentiation of flagellated swarmer cells to stalked cells. During this period, the bacterium ejects the flagellum, retracts the pili, synthesizes the stalk and holdfast structures, and initiates the replication of its DNA [6]. In the next stage, S, also called the pre-divisional stage, the DNA is completely replicated and the nucleoids are segregated [7]. This early pre-divisional compartmentalization produces differentiated cell poles [8]. In each of these micro-domains (as they are also known) a cell fate will arise; however it is still an open question how this biological process occurs. In the next stage, G2/M, the bacterium divides asymmetrically and the DNA becomes fully methylated [9]. As a result of these intricate processes, two cell types that differ morphologically and biochemically are generated [10]. The stalked cell presents stalk and holdfast structures that confer it the ability to remain attached to solid surfaces, and is the only one capable of replicating its own DNA [11]. A key regulator identified in the control of the cell cycle of *C. crescentus* is CtrA [12]. This transcription factor regulates approximately 100 genes, most of them dedicated to developing flagella and pili and to chemotaxis. CtrA binds to the *oriC* region of DNA, blocking the access of DnaA, and thus preventing the initiation of DNA replication [11]. The activities of CtrA and the regulators GcrA and DnaA constitute an interlinked regulatory network that is cell cycle-dependent and whose expression, at the mRNA and protein levels, timely oscillate during the different phases of the cycle [12], [13]. Other important molecular actors in this regulatory network are the methyl-transferase CcrM and the small CtrA-inhibitory protein SciP [14], [15]. An overview of the cell cycle in *C. crescentus* is illustrated in Figure 1. Given the differentiation phenotypes displayed by *C. Crescentus* and the nature of the regulatory network orchestrating the molecular interactions associated to this differentiation process, it is interesting to investigate how the dynamics of such interactions can reproduce the phenotypes observed in cell division and to what extend the interconnection between regulators and other involved molecules can account for (or reproduce) the appearance of the two phenotypes in the bacterium. In particular, the questions that we explore here are: Can the interconnection of the regulatory network and a simple description of its dynamics produce robust oscillatory behavior of the transcription factors during the stages of the cell cycle? How do network interactions generate the two micro-domains and the two cell types in *C. crescentus*?

An understanding of the principles on how bacterial differentiation occurs may have implications in other areas; for example, research on the origin of multicellularity, in which the asymmetric division of *C. crescentus* can give insight on basic differentiation processes and cell fate generation. Some mathematical models based on ordinary differential equations have been used to study the oscillations of the regulators associated to the cell cycle [16], [17], [18], revealing among other interesting facts the existence of a biological switch as the underlying mechanism operating in the asymmetric cellular division [19]. Nevertheless, the effect of the coupling between the transcriptional and signaling networks involved in the generation of the two cell types was not considered. More specifically, they did not take into account the influence of the proteolytic complex that degrades CtrA and the role of the transcription factor SciP in the dynamics of the networks.

Of course, several tightly regulated reactions must occur in order for the differentiation process to take place. For instance, the transcription of the regulator CtrA and its target genes, which highly depends on the concentration of phosphorylated CtrA. These reactions are influenced not only by the presence or absence of enzymes, but also by the rate at which the reactions can happen. What we intend to explore here, however, is the coherence between the dynamics and multi-stability displayed by a discrete logical model of a minimal network controlling asymmetric cell division and the observed phenotypes in the bacterial cell.

The justification for using this kind of modeling approach lies in its utility to explore or study questions of biological relevance at the network level. The process by which a model in general is constructed relies on assumptions and approximations. These may be for mathematical convenience (so that ‘simple’ models can be applied), but also because the modeling process puts a high value on simplicity, attempting to extract essential features of a system by working with the smallest possible number of concepts. According to this perspective, our model is supported by confronting experimental observations with the interpretations of attractor states obtained from simulations, as will be described later.

Towards our goal, we reviewed the relevant literature to gather all the known regulatory elements participating on the cell cycle and division processes of bacterium *C. crescentus*. We reconstructed the regulatory network and described the dynamics of the resulting network using a discrete formulation based on a generalization of the Boolean formalism for gene networks [20]. The choice of this formalism stems from the fact that we are interested on studying how the interactions and a simple dynamical description of changes of state in the regulatory network can generate the observed asymmetrical cell division. These models have been used to study multi-stability in the dynamics of large gene networks and the corresponding generation of cell fates or multiple phenotypes. Kauffman [21] and others [22], [23], [24] have successfully applied such models in real biological networks. Examples include the phage lambda, whose network dynamics has two steady states that correspond to lysis and lysogeny [22]; the regulatory network for flower development in *Arabidopsis thaliana* that has 10 stable states that correspond to its cellular fates [23]; the differentiation model of lymphocytes Th cells which display two phenotypes, each corresponding to attractors (dynamically stable patterns of expression) in the dynamical model [24].

## Data and Methods

### 1. Data acquisition

Regulatory pairwise interactions in the studied regulatory network were gathered from original literature (references to their PUBMED ID are cited in Table S1, sheet 1 in File S1). We performed a literature search and selected those reports that had information about gene regulation and its conditions in the cell cycle and asymmetric division of *C. crescentus*. We were careful in verifying that each regulatory interaction had direct experimental evidence. We also looked for information on the kind of regulation (positive or negative) for each interaction. When a protein promotes the transcription of a gene or activates another protein, it was considered a positive interaction. On the contrary, if a protein acts as a repressor, it was considered a negative interaction. In some cases, the regulators act dually (i.e., they act as both activators and repressors). The regulatory interactions include processes such as transcriptional regulation, methylation, phosphorylation/dephosphorylation, proteolysis and multi-protein complex assemble. The complete list of regulatory interactions gathered in this study is available as supporting information (Table S1, sheet 1 in File S1).

### 2. Reconstruction of the regulatory network (for cell cycle and cell fate in *C. crescentus*)

**Figure 2:**
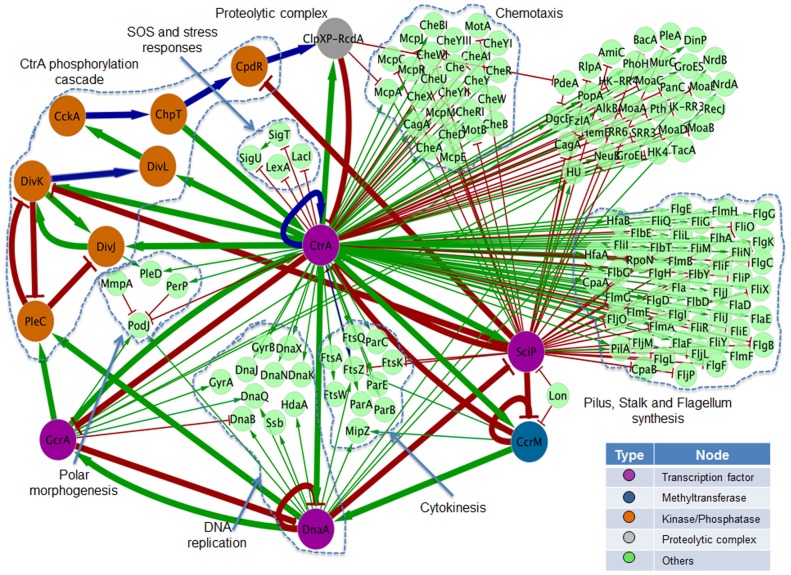
Regulatory network for the control of the cell cycle in *C. crescentus* (graph *G_1_*). Nodes represent genes/proteins and edges their regulatory interactions. These may be positives (green edges), negatives (red edges) or dual (blue edges). Purple nodes represent transcription factors; the blue node represents the methyl-transferase; the orange nodes correspond to kinases or phosphatases; and the gray node to the CtrA proteolytic complex. Larger nodes and thicker edges represent the core network that is modeled in this work (graphic *G_2_*).

A directed graph *G_1_* was drawn from the pairwise regulatory interactions obtained from the literature (see Figure 2). All the biological processes and proteins involved were included. The graph was drawn using Cytoscape [25]. In this abstract structure, the vertices or nodes represent genes or proteins and edges their regulatory interactions. Green edges indicate positive interactions, red edges stand for negative ones, and blue edges represent dual regulatory interactions. The graph contains all the proteins associated to the main transcription factors controlling the processes of DNA-replication and cell division, as well as additional cellular processes linked to the cell cycle, such as polar morphogenesis. Many of these proteins are involved in the development of the stalk or flagellum. The whole network *G_1_* is composed of 153 vertices (*V_1_* = 153) and 212 edges (*E_1_* = 212), supporting information (Table S1, sheet 1 in File S1).

#### 2.1. Reduction of the network to core regulators

**Figure 3:**
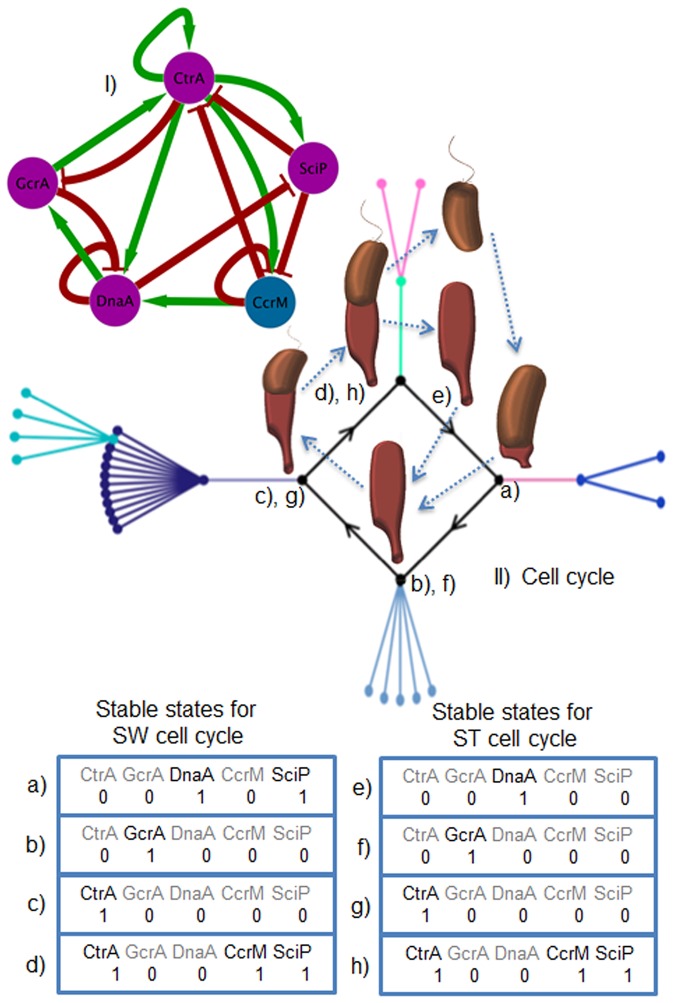
Cell cycle events and their correspondence with the steady states of the core regulatory *G_2a_*. The morphological changes for each cell type are illustrated through the cell cycle. I) Shows the regulatory network *G_2a_* that controls the cell cycle; II) Illustrates the state transitions and how all the states of the nodes reach a cyclic attractor. Subsections letters represent the states of the nodes in the two cell types (a, b, c, d for the attractor of the swarmer cell cycle and e, f, g, h for the attractor of the stalked cell cycle).

**Figure 4:**
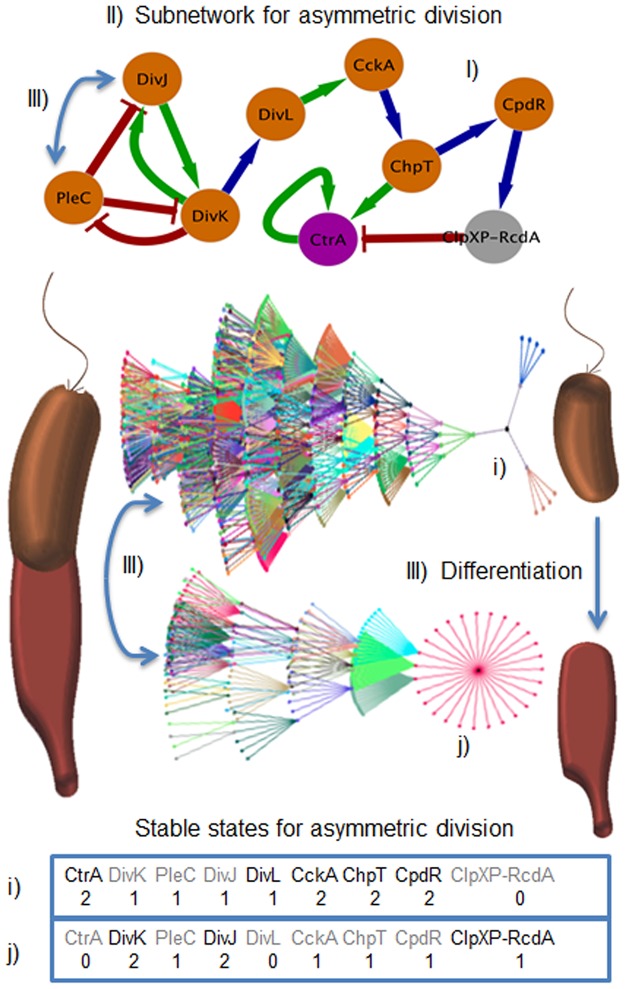
Cell fate emergence and differentiation processes. I) Phospho-proteolytic network *G_2b_*, which mediates cell fate and differentiation process. II) Transition state graphs show how all the states reach one of the two point attractors. III) Differentiation process switching from swarmer cell to stalked cell. a) This promoted by a change in the state of the kinase PleC by DivJ, b) In the model can be seen the switching effect form one attractor to the other. Subsections letters represent the states of the nodes in the two point attractors (i for the stable state of the micro-domain that will form the swarmer cell and j for the stable state of the micro-domain for the stalked cell).

We simplify the topology and the number of interactions in *G_1_* by removing non-regulatory nodes, i.e., those that do not regulate another node (green nodes in Figure 2). This criterion was chosen because non-regulatory nodes seem irrelevant for the network dynamics [26]. In this manner, we obtained the core network *G_2_* (see Figure 3.I and Figure 4.I). This is marked by thick edges on Figure 2. To further simplify the analysis of the network, *G_2_* was divided into two subnetworks: *G_2a_* and *G_2b_*, whose nodes operate in different processes and time scales. *G_2a_* is formed by transcriptional regulation and methylation processes (Figure 3.I) and *G_2b_* is constituted by a phospho-proteolytic pathway (see Figure 4.I). With regard to time-scales, these can be estimated on the order of ∼1–3 min for *G_2b_* and ∼1–100 ms for *G_2b_* [27]. The cross-interactions among elements of the subnetworks were treated in such a way that two well-separated subnetworks were obtained. Since the common node to these subnetworks was CtrA, it was designated as CtrA*_a_* and CtrA*_b_* in each subnetwork.

The reduced network *G_2_* has the minimum number of known elements that control the differentiation processes in the bacterium. All the proteins and genes constituting this network are listed in Table 1. The network *G_2_* has thirteen nodes (*V_2_* = 13) and twenty seven edges (*E_2_* = 27), see supporting information (Table S2, sheet 2 in File S1).

**Table 1:** Components (nodes) of the core regulatory network for cell cycle and cell fate (*G_2_*).

Protein/complex	Action in the cell cycle	Interactions in the *G_2_*	References PMID
CtrA	Master transcriptional regulator of the cell cycle. When it blocks *oriC* DNA region, the replication is inhibited. It promotes the asymmetric division and exists in high concentrations in the swarmer cell. Controls the transcription of genes for the stalk, pili, flagellum morphogenesis and chemotaxis (more than 100 regulated genes are known to date)	Transcription factor of GcrA (−), DnaA (+), CcrM (+), SciP(+) and its own promoter (+)	12445780
GcrA	Cell cycle transcriptional regulator. It regulates the transcription of genes for polar morphogenesis and DNA replication	Transcription factor of DnaA (−) and CtrA (+)	15087506
DnaA	DNA replication initiator protein. It regulates the transcription of genes for DNA replication and cytokinesis	Transcription factor of GcrA (+), SciP (−) and its own promoter (−)	16395331
CcrM	Adenine-specific methyl-transferase. It methylates genes which control the cell cycle	Methyl-transferase of the promoter of *dnaA* (+), *ctrA* (−) and its own promoter (−)	20472802
SciP	Small CtrA inhibitory protein. Through protein-protein interaction, it inhibits CtrA. It transcriptionally regulates genes (co-regulated with CtrA) for stalk, pili, flagellum morphogenesis and chemotaxis	Transcription factor of CtrA (−) and CcrM (−)	20472802
DivK	Cell division regulatory kinase. Through protein-protein interactions, it inhibits DivL when phosphorylated. In the unphosphorylated state, it promotes the binding of DivL to CcKA	Phosphorylates DivJ, dephosphorylate PleC and interacts with DivL (dual action)	7664732
DivJ	Sensory histidine kinase. Implicated in polar morphogenesis and in differentiation (by a not well understood process)	Phosphorylates DivK	20472802
PleC	Sensory transduction histidine kinase. Implicated in polar morphogenesis and differentiation (by a not well known process)	Dephosphorylates DIvK and DivJ	20472802
DivL	Tyrosine kinase. Promotes the auto-phosphorylation of CckA	Binds to CckA and promotes the auto phosphorylation of CckA	16547034
CckA	Cell-cycle histidine kinase. Phosphorylates and dephosphorylates ChpT	Phosphorylates/dephosphorylates ChpT	10199407
ChpT	Histidine phosphotransferase. Phosphorylates CtrA	Phosphorylates CtrA and phosphoprylates/dephosphorylates CpdR	20472802
CpdR	Two-component receiver protein. It inhibits the formation of the proteolytic complex	Promotes/inhibits the formation of the proteolytic complex ClpXP	20472802
ClpXP-RcdA	ATP-dependent protease complex and CtrA presenter protein. The assembled complex degrades CtrA	When this complex is formed, it degrades CtrA through RcdA (CtrA coupling protein)	16829582, 19747489

#### 2.2. Functional description of the transcriptional subnetwork *G_2a_*

As mentioned above, *G_2_* is made up of two coupled subnetworks, both linked by the vertex CtrA. In *G_2a_*, the regulator CtrA has dual transcriptional self-regulation (see figure 3.I). Its transcription is inhibited when the methyl-transferase, CcrM, methylates its promoter [28]. Another repressor of CtrA is SciP, which binds to CtrA and prevents it from binding to promoters, including the own promoter of CtrA [29]. The transcription factor GcrA promotes its own expression through a positive feedback loop [30], [31]. While CtrA inhibits the transcription of GcrA [30], DnaA promotes its expression [32]. The replication initiation protein DnaA is in turn subjected to other kinds of regulation; for example, when CcrM methylates the promoter of the gene *dnaA*, CtrA binds this region and promotes its transcription [33]. When the concentration of DnaA increases, this protein binds to its own regulatory region and represses its transcription [34]. Additionally, CtrA promotes the transcription of the gene *ccrM* [9]. The transcription of CtrA can only occur after the generation of the hemi-methylated state of the DNA (this state happens after the replication process when one strand in the double helix remains methylated and the other do not become methylated). When the concentration of CcrM increases, the protein methylates the second DNA strand, blocking the transcription of CtrA [35]. CtrA promotes the transcription of SciP and DnaA prevents it [15].

#### 2.3. Functional description of the phospho-proteolytic signaling subnetwork *G_2b_*

In the case of subnetwork *G_2b_* (see figure 4.I), CtrA activates the transcription of the sensory response regulator DivK [30]. Once produced, DivK is unphosphorylated and thus cannot sequester DivL, which binds to CckA and promotes the auto-phosphorylation of this kinase. When DivK becomes phosphorylated, either by the action of kinase DivJ or PleC (which can also act as a phosphatase) [36], it can bind to DivL and inhibit its activity [37]; as a consequence, CckA cannot auto-phosphorylate. In the phosphorylated state, CckA can phosphorylate the kinase ChpT [12]. However, when CckA is unphosphorylated, it can also act as a phosphatase dephosphorylating ChpT [38]. In the phosphorylated state, ChpT transfers a phosphate group to CtrA and activates it. Depending on the state of phosphorylation, ChpT can act as a kinase or a phosphatase of CpdR [39]. When CpdR is unphosphorylated, it mediates the formation of the proteolytic complex ClpXP, composed of the proteases ClpX and ClpP. These proteases together with the protein RcdA degrade CtrA [40], [41], [42].

### 3. Logical formalism for modeling the core network

As mentioned before, the central idea is to study how the interconnections and a simple description of the system dynamics, embodied in the reactions of the subnetworks, can account for the phenotypes observed in the bacterium being studied. To simulate the dynamics of the network, we used a discrete-logic multi-valued formalism, which is a generalization of the Boolean logic framework [43]. We choose this mathematical formulation due to its adequacy to describe additional node states (e.g., phosphorylation of CtrA) than those allowed by simple Boolean logic, which can only account for the presence or absence of regulators. In this formalism, the states of the nodes in a network can assume discrete numerical values; for example: 0, 1, …, *n*, depending on the states of other nodes, and this dependence is described by a regulatory function that maps, at each time step, the inputs (the states) from a set of nodes to the output of a particular node. Specifically, vertices in *G_2a_* take on two values, 0 and 1, which correspond to the cases of inactivity or activity, absence or presence of molecules, etc. In *G_2b_*, numerical values 0, 1 and 2 are used to represent the states of vertices corresponding to absence, presence and phosphorylation, respectively. The logical rules were defined using the operators OR, AND and NOT [20] (see Tables S3 to S16 in File S1). For example, in the case of *dnaA* whose transcription requires methylation by CcrM before being activated by the regulator CtrA, the assigned logical function is (CtrA) AND (CcrM). In another case, when GcrA inhibits the transcription of *dnaA*, a corresponding NOT function over the logical value of GcrA was used. For self-repression of *dnaA*, again a NOT function (DnaA) was assigned [33], [34]. Based on these criteria, we defined the following logical function for the value of DnaA in the next time step: DnaA*^t^* ^+1^ = (CtrA_a_ *^t^* AND CcrM*^t^*) AND (NOT GcrA*^t^*) AND (NOT DnaA*^t^*).

The dynamics of the studied subnetworks is described by an array of *n* discrete variables [σ_1_(*t*), σ_2_(*t*),… σ*_n_*(*t*)], where the variable σ*_i_*(*t*) represent the state of a particular node *i* at time *t*, which depends on the state of the *k* inputs (depicted by edges) coming from other nodes [σ*_i_*_1_(*t*), σ*_i_*_2_(*t*),…, σ*_i_*_k_(*t*)]. The evolution of the dynamics for each node in the system can be represented by the following equation: σ*_i_*(*t*+1) = *f_i_* (σ*_i_*_1_(*t*), σ*_i_*_2_(*t*),…, σ*_i_*_k_(*t*)); where *f_i_* is an appropriately chosen function that specifies a value for every possible combination of inputs to the node (refer to sheet 3, Tables S3 to S16 in File S1).

We employed GINsim [44] to simulate the dynamics of *G_2a_* and *G_2b_*. A synchronous strategy was used for updating the network states. All possible values that the vector [σ_1_(*t*), σ_2_(*t*),… σ*_n_*(*t*)] can assume define the state space of the network dynamics. A useful tool to depict the trajectories in state space followed by the network over time, starting from initial states (at time *t* = 0), is a transition state graph. This graph allows to portray particular states called attractors, which are dynamically stable patterns that the system tends to reach regardless the initial states. Attractors can mainly be divided in two classes: fixed point attractors, which do not have successive states, and limit cycle attractors whose states repeat after a period of time resulting in a cyclic pattern of states [45].

Apart from simulating dynamic states, we also performed perturbations to the vertices of subnetworks *G_2a_* and *G_2b_*, one at the time, by deleting genes (denoted as Δ) or by fixing a vertex to a specific value (resembling constitutive gene expression or over-expression) denoted as (+), and analyzed how the dynamics of the subnetworks changed under these scenarios (refer to File S1 for more details).

### 4. Dynamical regime of the networks

In general, the global behavior of networks can be classified into three regimes: chaotic, ordered, and critical [46]. In networks operating in the ordered regime, two randomly selected initial states, *S*_1_(0) and *S*_2_(0), that are separated from one another by a small Hamming distance, *h*(*t*) = |*S*_1_(*t*) - *S*_2_(*t*)|, will follow trajectories that rapidly converge on average, i.e., *h*(*t*) will approach zero as time tends to infinity. Both trajectories *S*_1_(*t*) and *S*_2_(*t*) will eventually settle down in an attractor, either a fixed point or limit cycle with a small length compared to the number of nodes in the network. In this sense, the network exhibits very stable dynamic behavior.

In the chaotic regime, the two randomly chosen initial states will produce trajectories that on average will deviate from each other over time and either end with high probability in two different attractors, or appear to ramble in state space. Networks in the chaotic regime do not produce stable behavior, as they quickly propagate perturbations in initial states. The critical regime lies on the limit between that of the ordered and the chaotic regime and confers to the network the ability to be robust to perturbations, but sensitive enough to state adaptations.

A common method for studying the propagation of perturbations in gene networks is based on the Derrida map [47]. In this approach, the degree of sensitivity to perturbations in the network is quantified by the slope *m* of a curve *M*(*h*(*t*)) that plots the evolution of the average Hamming distance over a large number of randomly picked initial states at each time step. The Derrida map provides a measure of the size of the perturbation at two consecutive time steps: *h*(*t*+1) = *M*(*h*(*t*)). The dynamical regime at which the network operates can be determined by computing the derivative *m* = *dM*(*h*)/*dh* at *h* = 0. As can be intuitively expected by observing the properties of *M*: when *m*<1, perturbations are absorbed (ordered regime); when *m*>1, perturbations are exacerbated (chaotic regime); and when *m* = 1, the network is partially sensitive to perturbations (critical regime). A Derrida map was generated for the network *G_2_*, and the change in its dynamical regime due to some perturbations (elimination of one node at a time) was also analyzed.

## Results

### Dynamics of the swarmer cell cycle

An overview of the cell cycle dynamics and the associated cellular events are shown in Figure 3.II. The transition state graphs and the attractor landscape resulting from the simulations of the network dynamics are also illustrated.

We found that all initial configurations of the transition state graph reach stable steady states. For *G_2a_*, the evolution of the states ends in a periodic attractor with four states (see Figure 3.II and Table S17 in File S1;). This cyclic attractor has correspondence with experimental evidence and simulates the oscillatory behavior of proteins CtrA, GcrA and DnaA during the cell cycle. In particular, using DNA microarrays of predicted open reading frames of the genome and a CtrA mutant (*ΔctrA401^ts^*), oscillations of the regulators at the level of mRNA have been observed throughout the cell cycle [35]. Similarly, by temporal protein immunopurification with specific antibodies (anti-CtrA and anti-GcrA), [47] observed the corresponding changes in protein concentrations during the stages of the cell cycle in *C. crescentus*.

We set up the initial state of the vertices in the cyclic attractor so that DnaA and SciP are active (Figure 3.II.a). In this state, DnaA is at high concentrations and could initiate the replication of DNA [48]. SciP represses the transcription of the genes involved in chemotaxis and development of pili and flagella [15], [27]. At this stage, the ejection of flagella occurs, and the synthesis of the holdfast and stalk structures starts; finally, the differentiation from the swarmer to the stalked cell begins.

In the next state, within the cyclic attractor, only GcrA is expressed (Figure 3.II.b). This protein promotes the expression of genes associated to the replication of DNA. In this state, the replication of DNA initiated by DnaA continues its elongation, and the chromosome begins to segregate to each of the cell poles. In the subsequent state, CtrA, which promotes the expression of genes for cell division, is the only protein that is active (Figure 3.II.c). The processes of DNA replication and chromosome segregation conclude, and a new round of DNA replication is inhibited due to the presence of hemi-methylated DNA strands [10]. This state is particularly characterized by the initiation of the asymmetrical cell division [49]. CtrA, as well as CcrM and SciP, remain active in the next state (Figure 3.II.d). In that state the full methylation of the DNA strands by CcrM occurs [8]. The asymmetric cell division takes place when CtrA, CcrM and SciP are maximally expressed (Figure 3.II.c and 3.II.d). This process is characterized by the formation of a divisional septum closer to one pole of the cell, which eventually generates the two cell types [50]. In this state, CtrA promotes the expression of proteins involved in the phosphorylation and proteolytic pathways [28].

It is known that the generation of spatial inhomogeneity inside cells is mediated mainly by ions and proteins gradients contributing to the generation of the so-called cellular micro-domains [51]. In *C. crescentus*, this is observed in pre-divisional cells, and is the product of a compartmentalization event and generation of a gradient of phosphate ions. Spatial micro-domains are observed near each of the two cell poles. The domain close to the pole that will develop the swarmer cell type is characterized by a high concentration of phosphate, in contrast to the other domain (corresponding to the stalked cell type) that has very low phosphate levels. The generation of cellular spatial heterogeneities associated to the micro-domains; and in particular, the generation of phosphate gradients and differential spatial localization of the kinases and phosphatases responsible for the asymmetric division of *C. crescentus* is supported directly (and indirectly) by experimental evidence [38], [52], [53] obtained by applying different techniques; for example, *in vitro* analysis of kinase, phosphatase, and phosphotransfer reactions confirmed the generation of two cellular conditions in which some regulatory proteins acted as kinases in one pole (when they were phosphorylated) whereas in the other pole, they acted as phosphatases when they were dephosphorylated [38].

Another source of cellular heterogeneity is produced by the polar localization of PleC and DivJ proteins. Using immunofluorescence and live cell microscopy techniques (in which the kinases were fused with fluorescent proteins), the specific location of these kinases in each of the cell poles could be observed [52], [53]. These experiments demonstrated the generation of the two differentiated micro-domains by the specific translocation of the kinases to each cell pole [52], [53].

PleC is anchored by PodJ to the pole that will give origin to the swarmer cell whereas DivJ is predominantly located on the pole that will give rise to the stalked cell type [52], [53]. Another important element in the generation of spatial inhomogeneity is the state of CtrA in the micro-domain associated to the swarmer cell type. In this micro-domain, phosphorylated CtrA (CtrA-P) is present at high concentration while in the other micro-domain CtrA is almost absent and mainly non-phosphorylated. This condition of CtrA is relevant, as in its active (phosphorylated) state, it binds to the *oriC* region of DNA blocking the initiation of replication and promoting the expression of genes that code for proteins involved in the formation of concentration gradients at each cell pole [10].

After simulating the dynamics of *G_2b_*, we found that all the initial states reach either of two point attractors (see Figure 4.II and sheet 4; Table S18 in File S1). Interestingly, the two point attractors are in correspondence with the states of the regulatory proteins in each of the two cell micro-domains near the poles described above. This could explain the functional partition of this regulatory network *G_2b_*, depending on the conditions inside the cell (phosphate concentration and protein location). The attractor corresponding to the state in which CtrA, Ccka, ChpT and CpdR are phosphorylated, and DivK and DivJ unphosphorylated, correspond to the micro-domain associated to the swarmer cell type (Figure 4.i). The attractor in which DivK and DivJ are phosphorylated, CckA, ChpT and CpdR are unphosphorylated, and the proteolytic complex is assembled, correspond to the micro-domain that will give rise to the stalked cell type (Figure 4.j).

### The stalked cell cycle and their dynamical differences with the swarmer cell cycle

As mentioned before, the dynamics of *G_2b_* generates two stable steady states. One corresponds to the swarmer cell and the other to the stalked cell type. In stalked cells, the proteolysis of CtrA changes the cyclic attractor of *G_2b_*. The states in the attractor corresponding to the stalked cell type are the only ones in which DnaA is expressed at maximum (Figure 3.II.e); in consequence, GcrA is maximally expressed too (Figure 3.II.f). The next state in the attractor is characterized by the expression of CtrA at its maximum level (Figure 3.II.g). In the following state, CtrA maintains its expression while CcrM and SciP become expressed (Figure 3.II.h). The difference between the cyclic attractor of the swarmer cell type and that of the stalked cell type is directly related to the expression of SciP and DnaA. In the former only DnaA is expressed, whereas in the latter both, DnaA and SciP, are maximally expressed. This might explain why SciP is expressed in a cell-type specific manner (in this case, in the swarmer cell) [27].

### Transition from swarmer to stalked cells

In the process of differentiation from a swarmer to a stalked cell many morphogenetic events occur. The ejection of the flagellum and pili, and the synthesis of the stalk are among the main events during this process. An important event that promotes differentiation is the replacement of PleC by DivJ. This replacement produces a change in the state of the kinase DivK in the swarmer cell, which triggers a phosphorylation-signaling cascade that promotes CtrA degradation [52]. In our discrete, logical model this switch behavior can be interpreted as changes in the states of PleC, DivJ and DivK, which induce a change at the system level from an attractor that corresponds to the micro-domain of the swarmer cell to the one corresponding to the stalked cell, (Figure 4.III). The state of nodes in this attractor makes biological sense since CtrA is degraded by the proteolytic complex (ClpXP-RcdA) in stalked cells. Our model also revealed that changes in the phosphorylation state of the kinases CckA and ChpT could lead to cell differentiation. These results, in agreement with a mathematical model recently developed [19], show that PleC (acting as a kinase or phosphatase) has a bi-stable response that propagates to the signaling network. Experimental observations [19] suggest the existence of a spatial replacement of PleC by DivJ, which is in agreement with our results. Changes in the phosphorylation state of the kinases in each of the cell poles and in their spatial localization were observed using immunofluorescence microscopy and *in vivo* phosphorylation assays [52]. From these observations, the spatial replacement of PleC by DivJ could be justified as a putative mechanism involved in the cellular differentiation process of *C. crescentus*.

### Simulating the dynamics of perturbed networks

We did not observe a significant change in the dynamics of *G_2a_* (Table 2) after simulating gene deletions in subnetworks *G_2a_* and *G_2b_* (refer to Table 2); nevertheless, perturbations in *G_2b_* (Table 3) might have more pronounced effects because this subnetwork turned out to be more sensitive to gene deletions. In spite of that, both subnetworks seem to be robust enough to maintain the cellular cycle, as well as other important processes in this bacterium (see Tables S19 to S22 in File S1, sheet 4).

**Table 2:** Dynamics of simulated gene deletions on *G_2a_*.

	Simulated deletion of nodes in the *G_2a_*	
Mutants	Effect	References PMID
Δ*ctrA_a_*	The cyclic attractor is perturbed. Only the states of DnaA and GcrA oscillate. This is in agreement with experimental data, where oscillations of DnaA are independent of the action of CtrA. A mutant of *ctrA* could not display asymmetric división	3143580
Δ*gcrA*	This mutation was experimentally lethal, but in our model this could not be observed	15087506
Δ*dnaA*	This mutation was lethal since the cells could not replicate their DNA, but the oscillations of CtrA are in agreement with experimental data	11309130
Δ*ccrM*	Experimental evidence shows that this deletion is lethal for the bacterium, in agreement with our model. This is because DnaA is not expressed	12234936
Δ*sciP*	It is predicted that the cycle is perturbed and change the state of expression of CtrA. This is in agreement with experimental evidence	22790399

	Simulated constitutive strains or over-expressed for the nodes in the *G_2a_*	
Constitutive strains	Effect	References PMDI
CtrA_a_(+)	The cycle is arrested to the point that only CtrA and SciP are expressed, causing the inhibition of DNA is replication, in agreement with experimental data	3143580
GcrA(+)	It could arrest the cell cycle because DnaA is not expressed. The rest of the genes are properly expressed, some experimental data suggest this finding.	15087506
DnaA(+)	The cell cycle is perturbed and DNA replication could be reinitiated repeatedly, since DnaA maintain its expression.	1309130
CcrM(+)	Cell cycle is arrested, as evident from the simulation. It explained why there is no expression of CtrA, GcrA, DnaA and SciP. It also caused a lethal effect in hemi-methylated DNA	12234936
SciP(+)	The cell cycle is arrested and evident cells are not viable, has correspondence with experimental data	20956288

**Table 3:** Dynamics of simulated gene deletions on *G_2b_*.

	Simulated mutants for the nodes in the *G_2b_*	
Mutants	Effect	References PMDI
Δ*ctrA_b_*	Makes cells unviable because only generates one attractor in which there is no phosphorylation of CtrA	3143580
Δ*divk*	Arrests cell cycle because only one stable state is formed, indicating that no proteolysis occurs to CtrA. The results are in agreement with experimental evidence	12237413
Δ*divJ*	For the mutant of *divJ*, it exhibits a single attractor, which is related to the swarmer cell type.	12852859
Δ*pleC*	For the mutant of *pleC*, it generates a normal phenotype, in agreement with experimental data	12852859
Δ*divL*	Causes a change in the phosphorylation state of CtrA, but not in proteolysis, in agreement with experimental evidence	17827294
Δ*cckA,* Δ*chpT*	There is neither phosphorylation of CtrA nor assembly of the proteolytic complex causing a degradation of CtrA, in agreement with experimental evidence	12603734, 10199407
Δ*cpdR*	Shows no evident alterations, only the fact that the proteolytic complex is not assembled and there is no CtrA proteolysis	16829582
Δ*clpXP-rcdA*	There is not proteolysis of CtrA, but there is proper phosphorylation, in agreement with evidence	19747489, 12445780

	Simulated constitutive strains or over-expressed for the nodes in the *G_2b_*	
Constitutive strains	Effect	References PMID
CtrA_b_(+)	There is no proteolysis of CtrA	3143580
DivK(+), DivJ(+), PleC(+)	Promote the presence of only one cell type and no cell division. The cycle is arrested, some experimental data suggest these findings	12852859
DivL(+),CckA (+), ChpT(+), CpdR(+)	Prevents the proteolysis of CtrA, and the division of cells. The cell cycle is arrested. There is not experimental data to compare	-----
ClpXP-RcdA(+)	There are no CtrA phosphorylation and no CtrA expression, causing a lethal effect. There is not experimental data to compare	-----

### Dynamical regime of network *G_2_*

**Figure 5:**
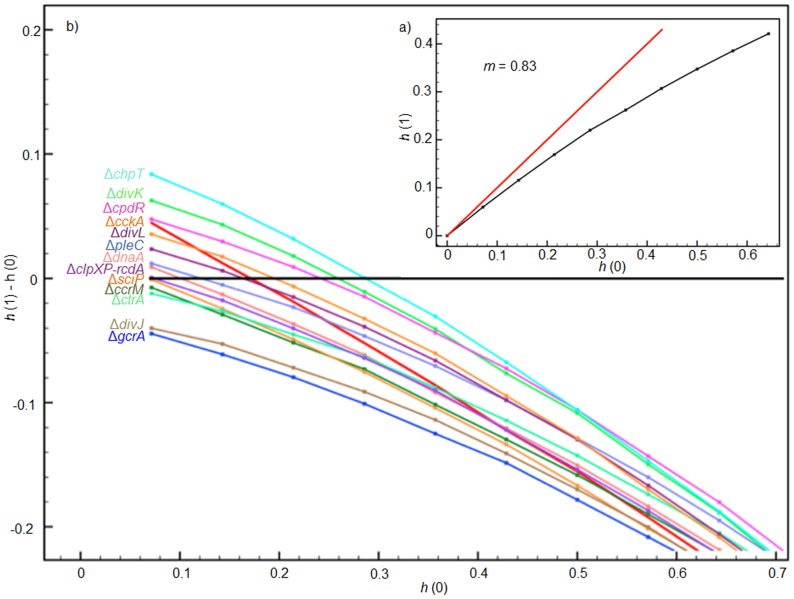
Derrida map for determination of dynamical regime. The network operates in the ordered regime close to the critical regime. a) Derrida map of the network *G_2_*, b) Derrida map of the network where a node was eliminated one at a time (indicated by Δ).

We found that the studied network operate in the ordered regime near criticality (*m* = 0.83), as can be seen in Figure 5.a Figure 5.b shows how the elimination of vertices changes the dynamics of the entire network *G_2_*, driving the network to a chaotic regime. Only deletions of genes *gcrA*, *divJ* and *sciP* (referred to as Δ*gcrA*, Δ*divJ* and Δ*sciP*) take the network to a more ordered regime. The operation of the regulatory network *G_2_* near the critical regime may explain why the bacterium, even when subjected to constant changing environments, is able to maintain its characteristic cell-cycle and asymmetric division.

## Discussion

A previous sketch of the coupled networks *G_2a_* and *G_2b_* presented here has been already outlined by McAdams and Shapiro [13], [53]. They introduced the notion of a core engine with a hierarchical activity of regulators controlling the cell cycle. They also suggested the existence of a phosphorylation-signaling pathway mediating asymmetric cell division and controlling the phosphorylation states of CtrA. In agreement with these observations (proposals), we constructed a discrete dynamical model for cell fate emergence based on the integration of the network *G_2a_* (which regulates the cell cycle) with a signaling network G*_2b_* (which acts as a phosphorylation-signaling pathway).

Our model provides results that might validate the function of the regulatory subnetwork *G_2b_* in the control of the whole cell cycle. We propose that the proteolytic complex in *G_2b_*, given by the association of ClpXP and RcdA (the CtrA presenter protein), could also be integrated in the phosphor-signaling pathway. CtrA, a central regulator in the network studied here, exhibits discrete oscillatory behavior during the different phases of the cell cycle in *C. crescentus*. We found that the architecture of the *G_2b_* produces oscillations in the activity of the regulatory proteins. Our discrete modeling approach describing the kinetics of synthesis, degradation and activity of the regulators reproduces biological observations on the role of the networks. From the nature of our model and simulations, we speculate that it is possible for the regulators in the network to act in a synchronized manner. Several models [have tried to explain or have been proposed] to explain the control of the cell cycle and the oscillatory behavior of the regulatory proteins associated to this process [16], [17]. However, these models did not consider how network dynamics give rise to the formation of the two known cell types in *C. crescentus*. Furthermore, they do not explain the cell type-dependent expression of SciP and its relevance in the regulation of cell cycle, as has been described in the recent literature [27]. Our model illustrates the putative mechanisms for the formation of the two cell types and integrates the activity of the protein SciP in the regulation of the cell cycle; more specifically, our model shown why SciP expression is performed only in the swarmer cell type.

Another model [19] has suggested that PleC could have bi-stable behavior and that could lead to the formation of the cell types. This proposal is in agreement with our finding of two stable states in the phospho-proteolysis network and the formation of the micro-domains. Moreover, we took also into account the role of the proteolytic complex of CtrA (ClpXP-RcdA), and explained how an initial phosphorylation state of the kinases, or the proteases in the G*_2b_* could lead to the formation of each cell type. We developed a dynamical description of proposals made in the literature about the emergence of the two cell types [35], [54].

The dynamical regime in which the network operates is near the critical regime. Biological networks have been shown to operate in this regime or very close to it [55]. It has been argued that such networks are adaptive to changes, but robust enough to perturbations [56].

Several requirements have been stated for an organism to be considered multicellular, such as a complex developmental program, robust differentiation, and emergence of germ-line cells [57]. Some of these traits are found in *C*. *crescentus*. Because of this, in order to under stand some steps in the origin of multicellular life forms it is important to study the dynamics of cell cycle and the emergence of cell fates. Our dynamical model could shed light on the formation of differentiated cells from a polarized cell. It has also been proposed that there exist three types of multicellularity. One of them is characterized by the division and generation of two different cell types, and only one of these is capable of generating both cell types [58]; for example the asymmetric division of *C. crescentus*. Using our model we could explain this phenomenon and contribute to the understanding of the origin of multicellular organisms.

## Conclusions

The model of cell cycle, differentiation and asymmetric cell division in *C. crescentu*s presented here was reconstructed from original literature and considered the assumptions described in previous models. By using a discrete formalism to model the dynamics of the network, it was possible to explain the operation of the whole network, as reconstructed from individual events collected from the literature. The network exhibits oscillatory behavior of the regulators during the cell cycle and remarks their temporal activity. This temporal activity could be explained in the context of spatial micro-domains formed in the cell under precise conditions. The phosphorylating and proteolytic pathways, which converge in the global regulator CtrA, lead to the generation of the two cell types.

On the one hand, what we currently know about the regulatory network that controls the cell cycle in *C. crescentus* is the fact that it directs a robust and complex process, able to buffer perturbations on the network without propagating dysfunction. On the other hand, the relatively long cascade of kinases and proteolytic proteins makes the network sensitive enough to respond to multiple environmental conditions. This work contributes to understanding the regulatory mechanisms that operate at the core of the asymmetric cell division process in *Caulobacter crescentus*.

## Supporting Information

File S1 Sheet 1: Complete network *G_1_* with the complete list of genes/proteins in associated to the cell cycle control in *C. crescestus*; the biological function, type of interaction and kind of regulation are described for each node, including the PubMed ID (PMID) of their experimental evidence. Sheet 2: List of genes/proteins in the reduced network *G_2_*. The biological function, type of interaction and the kind of regulation are described for each node, including the PubMed ID (PMID) of their experimental evidence. Sheet 3: Logical functions and their rules as well as their respective biological descriptions. Sheet 4: Simulation of the network dynamics. Table S1: Complete network (G1) of regulators and their interactions associated to the control of the cell cycle in *C. crescentus*. Table S2: Reduced network (G2) of proteins and their interactions for the cell cycle control in *C crescentus*. Table S3: Logical function for the expression or inhibition of CtrA. Table S4: Logical function for the expression or inhibition of GcrA. Table S5: Logical function for the expression or inhibition of DnaA. Table S6: Logical function for the expression or inhibition of CcrM. Table S7: Logical function for the expression or inhibition of SciP. Table S8: Logical function for the phosphorylation or degradation of CtrA. Table S9: Logical function for the phosphorylation or dephosphorylation of DivK. Table S10: Logical function for the phosphorylation or dephosphorylation of PleC. Table S11: Logical function for the phosphorylation or dephosphorylation of DivJ. Table S12: Logical function for the activation of DivL. Table S13: Logical function describing the activation of inhibition of CckA. Table S14: Logical function for the phosphorylation or dephosphorylation of ChpT. Table S15: Logical function for the phosphorylation or dephosphorylation of CpdR. Table S16: Logical function for the assembly or unassembly of the ClpXP-RcdA complex. Table S17: Cyclic states for regulators of the cell cycle network. Table S18: Punctual attractor states in the dynamics of the phospho-proteolytic network. Table S19: Steady states after deleting genes *ctrA*, *gcrA*, *dnaA*, *ccrM*, and *sciP* (one at a time) in the cell cycle network *G_2a_*. Table S20: Steady states after fixing the values of CtrA, GcrA, DnaA, CcrM, and Scip in the cell cycle network *G_2a_*. Table S21: Steady states after deleting genes *ctrA*, *divK*, *pleC*, *divJ*, *divL*, *cckA*, *chpT*, *cpdR* and the complex *clpXP*-*rcdA* in the phospho-proteolytic network *G_2b_*. Table S22: Steady states after fixing the values of CtrA, DivK, PleC, DivJ, DivL, CckA, ChpT, CpdR, and ClpXP-RcdA in the phospho-proteolytic network *G_2b_*.
(XLSX)
